# Selection of pre-trained weights for transfer learning in automated cytomegalovirus retinitis classification

**DOI:** 10.1038/s41598-024-67121-7

**Published:** 2024-07-10

**Authors:** Pitipol Choopong, Worapan Kusakunniran

**Affiliations:** 1 Department of Ophthalmology, Faculty of Medicine Siriraj Hospital, Mahidol University, Bangkok, Thailand; 2 Present Address: Faculty of Information and Communication Technology, Mahidol University, Nakhon Pathom, Thailand

**Keywords:** Machine learning, Retinal diseases, Viral infection, Computer science

## Abstract

Cytomegalovirus retinitis (CMVR) is a significant cause of vision loss. Regular screening is crucial but challenging in resource-limited settings. A convolutional neural network is a state-of-the-art deep learning technique to generate automatic diagnoses from retinal images. However, there are limited numbers of CMVR images to train the model properly. Transfer learning (TL) is a strategy to train a model with a scarce dataset. This study explores the efficacy of TL with different pre-trained weights for automated CMVR classification using retinal images. We utilised a dataset of 955 retinal images (524 CMVR and 431 normal) from Siriraj Hospital, Mahidol University, collected between 2005 and 2015. Images were processed using Kowa VX-10i or VX-20 fundus cameras and augmented for training. We employed DenseNet121 as a backbone model, comparing the performance of TL with weights pre-trained on ImageNet, APTOS2019, and CheXNet datasets. The models were evaluated based on accuracy, loss, and other performance metrics, with the depth of fine-tuning varied across different pre-trained weights. The study found that TL significantly enhances model performance in CMVR classification. The best results were achieved with weights sequentially transferred from ImageNet to APTOS2019 dataset before application to our CMVR dataset. This approach yielded the highest mean accuracy (0.99) and lowest mean loss (0.04), outperforming other methods. The class activation heatmaps provided insights into the model's decision-making process. The model with APTOS2019 pre-trained weights offered the best explanation and highlighted the pathologic lesions resembling human interpretation. Our findings demonstrate the potential of sequential TL in improving the accuracy and efficiency of CMVR diagnosis, particularly in settings with limited data availability. They highlight the importance of domain-specific pre-training in medical image classification. This approach streamlines the diagnostic process and paves the way for broader applications in automated medical image analysis, offering a scalable solution for early disease detection.

## Introduction

The retina, a crucial component of the eye, perceives light and transforms it into neural signals for brain interpretation, playing a pivotal role in human vision. Any damage to the retina leads to unrecoverable loss of sight. Cytomegalovirus retinitis (CMVR) is an infectious disease affecting all layers of the retina. Retinal cells are vulnerable and permanently destroyed by the virus. Consequently, the infection leads to severe retinal damage and permanent blindness. The infection usually occurs in patients with low immune status, such as HIV-infected, cancer, and immunosuppressed patients. The global prevalence of HIV-related CMVR is high, especially in low- and middle-income countries in Asia, where the pooled prevalence is 14%^1^. In addition, the incidence of CMVR in non-HIV individuals is increasing according to malignancies and the usage of immune suppressive therapies^2^. Therefore, the screening fundus examination is needed routinely every three months to prevent blindness in these high-risk patients^3^. However, regular eye screening in low-resource settings, such as rural areas in developing countries, is challenging^4^.

The standard screening procedures for eye disease prevention include a visual acuity test, intraocular pressure check, eye examination, and retinal examination in patients with high risks of developing disease by ophthalmologists or optometrists. However, ophthalmic personnel are rarely available, and commute services are limited in rural districts^5,6^. Given these challenges, particularly in remote and resource-limited settings, there is a pressing need for innovative approaches like telemedicine and automated detection systems to ensure timely and effective diagnosis of CMVR.

There were attempts to overcome these limitations using telemedicine^7–10^. The retinal photographs were taken at local hospitals and later read locally by trained paramedics or ophthalmologists at the reading centre. The results were comparable to expert reading but with high variability. In addition, this operation consumes time to train reading staff and money to set up a reading centre. Given these limitations, new solutions are needed.

Automatic detection and classification in ophthalmology through retinal image analysis have increased interest. Early techniques were based on handcrafting methods, which were laborious and time-consuming^11^. With the growth of Deep Learning technology, particularly convolutional neural networks (CNN), the technique has moved forward to construct algorithms to analyse retinal images automatically^12,13^. With successful examples of using CNN to classify diabetic retinopathy (DR) and other retinal diseases^14^, applications of the CNN models should help early detection of retinal diseases such as CMVR.

However, developing a suitable automated model for a particular retinal disorder requires a large dataset with accurate ground truth. Different algorithms with varying accuracies for other retinal diseases have been proposed. Consensus has yet to be made on the best technique to build the model. Two previous articles compared several state-of-the-art CNN models to detect CMVR. Srisuriyajan P et al. found that VGG16 showed the best performance, while Du KF et al. achieved the best result using Inception-Resnet-v2^15,16^.

Despite a suitable model algorithm, the appropriate number of datasets is also mandatory. Conventional CNN development usually requires more than a million images to build a high-performance model. This requirement is often limited in medical imaging fields, particularly in CMVR images, where the resource is scarce. Transfer learning (TL) is a common approach to deal with CNN modelling on small datasets, particularly medical image datasets^17,18^. The process works by using the weights from a model developed from other related source domains where the training data is sufficient and further refining the model weights for a different target domain operating on the same or similar task. For image classification, low-level features such as dots, edges, and straight and curved lines are shared among all images. Therefore, standard TL uses the low-level feature weights from the model trained primarily on large natural image datasets. The required number of pictures on the target dataset can be remarkably reduced to hundreds or thousands of images with proper pre-trained model weights.

The most common source weight for the classification task is from the training on the ImageNet dataset, which consists of common natural images^19^. However, it can be argued that the characteristics of those images are far different from retinal images. For instance, retinal images have much higher levels of red and green channels and are consistent in anatomical structures compared to natural images. Therefore, the idea of sequential TL, transferring weights from the very large dataset of natural images as the source domain to a large medical image dataset of the intermediate domain and then to the final small medical image dataset of the task domain, was introduced.^20,21^

Nevertheless, previous studies showed controversies in the selection of pre-trained weights. MRH Taher et al. found that domain-adapted pre-trained models outperform the corresponding ImageNet and in-domain models for the classification tasks on chest X-ray images^20^. In contrast, Y Wen et al. demonstrated that pre-training from ImageNet showed better performance^21^. Moreover, the benefit was demonstrated only in large-dataset experiments^20^. With a limited number of CMVR photos in our dataset, in this article, we investigated the comparison of our model performances on the diagnosis of CMVR using DenseNet121 as a backbone under three TL approaches with weights trained from ImageNet, retinal images (APTOS2019 dataset), and chest X-ray images (CheXNet dataset).

## Material and methods

We collected the study's dataset from the Department of Ophthalmology at Siriraj Hospital, Mahidol University, with Siriraj Institutional Review Board approval (SiIRB#552/2015). The research was conducted according to the 2002 version of the Declaration of Helsinki. Informed consent was obtained from all subjects for unidentifiable use of their images.

### Dataset and preprocessing

The dataset for this study was collected over a decade, from January 2005 to December 2015, at the Department of Ophthalmology, Siriraj Hospital. We utilised Kowa VX-10i and VX-20 fundus cameras to capture the retinal images. These images, in RGB format with a resolution of 1280 × 960 pixels, were saved in JPEG format. While most images focused on the central retinal areas, some also included lesions in the far peripheral retina.

For our analysis, the images were meticulously screened for clarity and readability. Subsequently, they were classified into two categories: CMVR and Normal images. The CMVR diagnosis was confirmed through clinical examination or molecular identification of CMV in the ocular fluid. The three main clinical presentations of CMVR are fulminant (classic) form: sectoral, full-thickness, yellow-whitish, retinal infiltrations with retinal haemorrhages; indolent (granular) form: peripheral, granular, whitish retinal opacity; and frosted branch angiitis (perivascular) form: perivascular infiltrations without retinal involvement. All CMVR images included one or a mixture of these characteristics. Normal photos were sourced from patients who underwent routine eye screenings, primarily for DR and exhibited no retinal abnormalities.

Our dataset comprised a total of 955 images from 94 patients, with 524 categorised as CMVR and 431 as Normal. The patient’s demographics and characteristics are demonstrated in Table 1. The dataset was pre-partitioned for training, validating, and testing purposes, ensuring a comprehensive evaluation of the model's performance. To enhance the robustness of our training dataset, we employed various image augmentation techniques, including flipping, mirroring, brightness adjustment, shifting, rotation, and zooming. Specific augmentation parameters included a width shift and height shift of 0.5, a rotation range of 90 degrees, horizontal and vertical flips, a brightness range from 0.1 to 2.0, and a zoom range of 0.25.

**Table 1:** Demographic data, characteristics, and diagnoses of patients.

	CMVR (n = 28)	Normal (n = 66)	Total (n = 94)
Average age in years (SD)	38.8 (8.3)	40.4 (21.8)	39.9 (18.8)
Male (%)	15 (53.6)	25 (37.9)	40 (42.6)
HIV infected (%)	26 (92.9)	18 (27.3)	44 (46.8)
CMVR phenotypes (%)			
Fulminant form	10 (35.7)		
Indolent form	6 (21.4)		
Perivascular form	2 (7.2)		
Mixed form	10 (35.7)		
Retinal photos in training dataset	363	295	658
Retinal photos in validating dataset	109	78	187
Retinal photos in testing dataset	52	58	110

### CNN model

**Figure 1:**
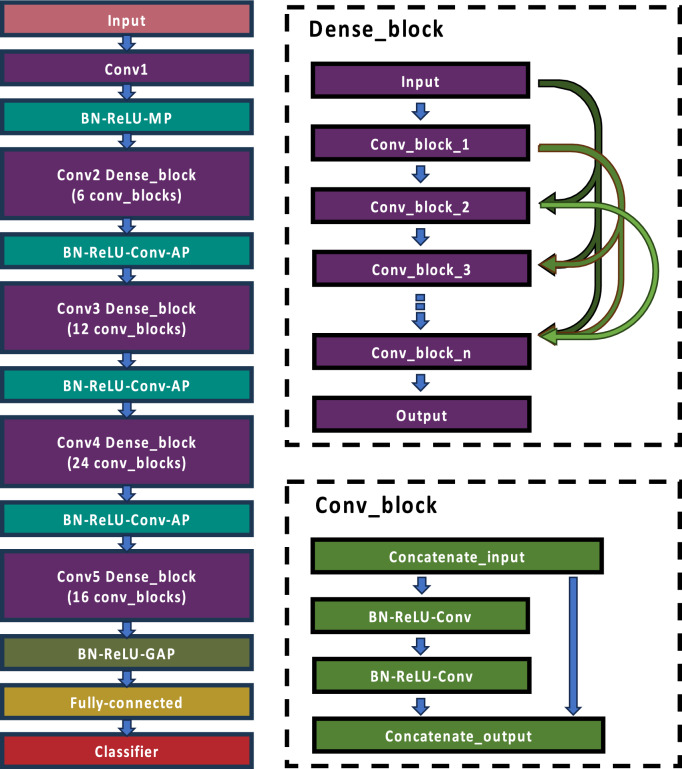
A schematic diagram of DenseNet121 architecture.

Our study employed DenseNet121 as the foundational model to assess various TL strategies. DenseNet, depicted in Fig. 1, was introduced by Huang et al. in 2017^22^. This architecture is a derivative of ResNet and is distinguished by its utilization of shortcut connections, enabling each layer to be directly connected to every other layer. In DenseNet, the input to each convolutional layer comprises the aggregated feature maps from all preceding layers, and its output is fed into all subsequent layers. This unique approach of feature map concatenation enhances DenseNet's computational and memory efficiency.

The DenseNet architecture begins with an initial sequence of a 7 × 7 convolution, batch normalization, ReLU activation, and max pooling. This is followed by four dense blocks (Dense_blocks) and concludes with global average pooling, fully connected, and classification (softmax) layers. Each dense block is interspersed with a transitional layer consisting of batch normalization, ReLU activation, a 1 × 1 convolutional layer, and average pooling. Within each Dense_block is a series of Conv_blocks, which are combinations of 1 × 1 and 3 × 3 convolutional layers. The specific number of Conv_blocks varies depending on the DenseNet model variant. DenseNet121, in particular, contains a total of 120 convolutional layers.

**Figure 2:**
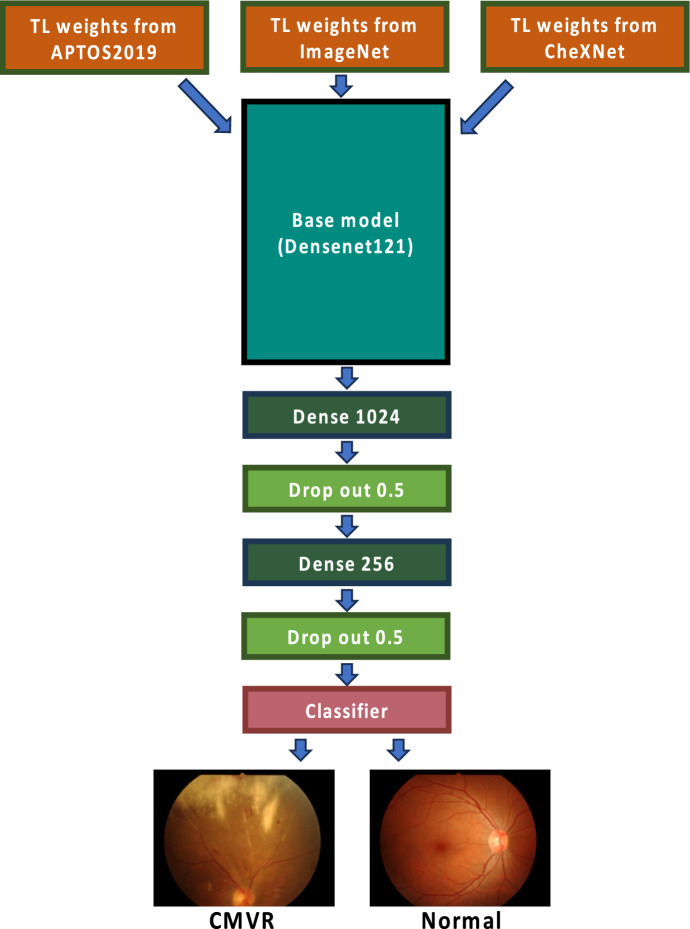
Proposed method to evaluate the transfer learning strategies.

In our experiments, we modified the network by replacing the original top fully connected and classification layers with two new fully connected layers, a 50% dropout layer, and a 2-class classification layer, as illustrated in Fig. 2.

### Transfer learning

We compared different pre-trained weights used in TL. Since there is no established consensus in the selection of weights for retinal image datasets, we explored the feature weights trained from 3 sources: ImageNet, sequentially trained from ImageNet to APTOS2019, and sequentially trained from ImageNet to CheXNet datasets. All three weights are transferred to classify our retinal images via different fine-tuning levels (Fig. 2).

- ImageNet weight is a CNN algorithm trained on the ImageNet dataset from the ImageNet Large Scale Visual Recognition Challenge (ILSVRC) to classify and localise 1,000 object classes^23^. The dataset contains over one million colour images of natural daily lives, such as cats, dogs, and vehicles. Many state-of-the-art CNN models are pre-trained on this dataset, for instance, VGG, ResNet, Inception, and DenseNet. The pre-trained ImageNet weights were obtained via the Keras application^24^.
- A sizeable retinal image dataset is from the Asia Pacific Tele-Ophthalmology Society (APTOS) 2019 Blindness Detection competition provided by APTOS on the Kaggle website^25^. The competition aims to classify the DR images. The dataset contains five classes of 3,662 full-colour retinal fundus images ranging from no DR, mild DR, moderate DR, severe DR, and proliferative DR. Briefly, the model was trained on the APTOS2019 dataset using transfer learning from ImageNet under the DenseNet121 model. The final weights were then collected for our experiment from the Kaggle website^26^.
- CheXNet weights are results from a deep learning algorithm that can detect and localise 14 kinds of diseases from chest X-ray images^27^. Based on DenseNet121 and ImageNet transfer learning, the model is trained on the ChestX-ray14 dataset from the National Institute of Health, containing 112,120 frontal view X-ray (black and white) images from 30,805 unique patients^28^. The pre-trained weights were obtained through the GitHub website^29^. The chest X-ray images displayed some similarities to retinal images as they are medical pictures with stereotypic and spatial preservation. They are more abundant and publicly available compared to the retinal image dataset, which may serve as a potential intermediate source for sequential TL.

First, we evaluated the depth of fine-tuning methods among the three pre-trained weights in 2-class (CMVR vs Normal) identifications. After determining the best depth, we further compared the diagnostic performance of the three best models. We assumed the sequential TL from a similar target domain would offer the best result. The statistical analyses were performed using one-way analysis of variance (ANOVA). We considered p-values < 0.05 as statistically significant. All analyses were conducted using SPSS version 18.0 (SPSS, Chicago, IL, USA).

### Performance evaluation

The experiments were performed on an Intel^®^ Core™ i9-10940X CPU @ 3.30 GHz with 252 GB of RAM and an NVIDIA GeForce 3090 12 GB for 100 epochs. The training lasted 8 h. The best and 10-time average performances were assessed. We adopted many performance indices for model evaluation. Accuracy and Loss are two primary metrics considered during the model training. Accuracy is the proportion of correct prediction (where the predicted values are the same as actual values) over the total predictions. Loss is a continuous variable displaying the uncertainty of how much the prediction varies from the true value. For the classification task, the default loss function is cross-entropy. The optimiser will learn and adjust the weights in each iteration to reach the maximal accuracy and minimal loss in the model development. The formulae for accuracy and cross-entropy function were defined as:

1 $$Accuracy= \frac{No. \, of \, correct\, predictions}{Total \, No.\, of\, predictions}$$

2 $$Cross\, entropy= -\sum_{i=1}\sum_{j=1}{y}_{i,j}\text{log}\left({p}_{i,j}\right)$$

where ${y}_{i,j}$ denotes the true value i.e. 1 if sample i belongs to class j and 0 otherwise and ${p}_{i,j}$ denotes the probability predicted by the model of sample i belonging to class j.

For model evaluation, we used a confusion matrix to visualise and summarise the performance of a classification algorithm. It represents counts of predicted and actual values as True Positive (TP), True Negative (TN), False Positive (FP), and False Negative (FN). The performance indicators from the confusion matrix are sensitivity (recall), specificity, positive predictive value (precision), accuracy, and F1 score.

3 $$Sensitivity\, \left(Recall\right)=\frac{TP}{TP+FN}$$

4 $$Specificity=\frac{TN}{FP+TN}$$

5 $$Positive\, predictive\, value \left(Precision\right)=\frac{TP}{TP+FP}$$

6 $$Accuracy=\frac{TP+TN}{TP+FP+TN+FN}$$

7 $$F1 \, score=\frac{2TP}{2TP+FP+FN}$$

### Activation maps

For a better understanding of the model activities, class activation heatmaps were produced to identify the predictive areas on the retinal image. We applied Class Activation Mapping (CAM) architecture for this purpose^30^. In brief, CAM works by modifying the structure of the CNN model, particularly towards the end of the network. It replaces fully connected layers with global average pooling layers, followed by a classification layer. This alteration allows for the generation of maps using the weights of the classification layer. These maps are essentially heatmaps that highlight the influential areas of the input image for the classification task. Then, the heatmaps were upscaled and applied to the original image. We presented class activation heatmaps from models pre-trained with the three feature weights to identify hot spots triggering the classification.

## Results

In the fine-tuning state, we found that training from Convolutional block 4 (Conv4) resulted in the best performance among the three approaches. (Table 2) The three best strategies were TL with APTOS2019 weights trained from Conv4 (accuracy 0.99, loss 0.04), ImageNet weights trained from Conv4 (accuracy 0.98, loss 0.06), and CheXNet weights full-trained (accuracy 0.97, loss 0.09). There were statistically significant accuracy differences among the three models, as indicated by the ANOVA test (p < 0.001). The post-hoc analyses demonstrated that all pairs were different. (APTOS2019 vs ImageNet, p = 0.02; APTOS2019 vs CheXNet, p = 0.0001; ImageNet vs CheXNet, p = 0.039).

**Table 2:** Average validating accuracy and loss of 2-group (CMVR vs normal) classification from transfer learning using pre-trained weights of APTOS2019, ImageNet, and CheXNet on DenseNet121.

Training strategies	APTOS2019		ImageNet		CheXNet	
On DenseNet121	Mean accuracy (SD)	Mean loss (SD)	Mean accuracy (SD)	Mean loss (SD)	Mean accuracy (SD)	Mean loss (SD)
Full train	0.9082 (0.045)	0.2299 (0.07)	0.9269 (0.022)	0.3681 (0.407)	0.9742 (0.004)	0.0968 (0.011)
From Conv2	0.9501 (0.025)	0.1745 (0.044)	0.9554 (0.012)	0.1444 (0.029)	0.9697 (0.006)	0.1095 (0.013)
From Conv3	0.9644 (0.006)	0.099 (0.008)	0.9635 (0.01)	0.0997 (0.022)	0.9688 (0.01)	0.1109 (0.013)
From Conv4	0.9911 (0.006)	0.0439 (0.006)	0.9822 (0.006)	0.0601 (0.002)	0.9653 (0.006)	0.1229 (0.015)
From Conv5	0.9751 (0.004)	0.0936 (0.009)	0.9679 (0.003)	0.1034 (0.012)	0.9483 (0.011)	0.2055 (0.014)
No train	0.9653 (0.003)	0.1571 (0.003)	0.9242 (0.012)	0.2375 (0.018)	0.8655 (0.019)	0.4389 (0.03)

*CMVR* cytomegalovirus retinitis, *APTOS* the Asia Pacific Tele-Ophthalmology Society, *SD* standard deviation, *Conv* convolutional block.

**Figure 3:**
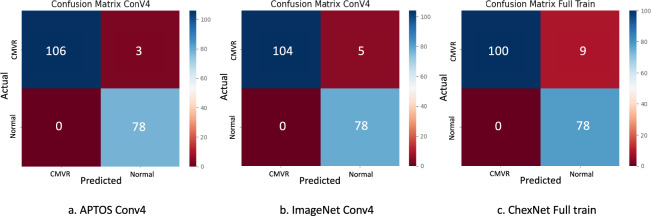
Confusion matrices generated with Python codes demonstrated validating performance of models with transfer learning weights from (**a**) APTOS2019 Conv4, (**b**) ImageNet Conv4, and (**c**) CheXNet full train; *Conv* convolutional block, *APTOS* the Asia Pacific Tele-Ophthalmology Society, *CMVR* cytomegalovirus retinitis.

**Figure 4:**
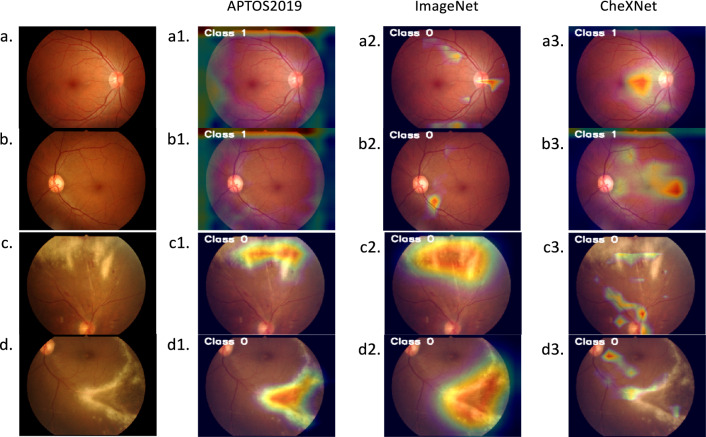
Original images of normal retina (**a,b**) and CMVR (**c,d**) images and heatmaps (using Class Activation Mappings^30^) overlayed images from models with transfer learning weights from APTOS2019 conv4 (**a1–d1**), ImageNet conv4 (**a2–d2**), and CheXNet full train (**a3–d3**); *Conv* convolutional block, *APTOS* the Asia Pacific Tele-Ophthalmology Society, *CMVR* cytomegalovirus retinitis.

The confusion matrices and performance indicators of the three best models on the validating dataset are shown in Fig. 3 and Table 3. The performance accuracies were 0.98, 0.97, and 0.95 for models with pre-trained weights from APTOS2019 conv4, ImageNet conv4, and CheXNet full train, respectively. Samples of Class activation maps overlayed images were displayed in Fig. 4. In a similar way, Table 4 demonstrates the model’s performance indicators analysed from the testing dataset. Their accuracies improved to 0.99, 0.99, and 0.98, respectively.

**Table 3:** Performance indicators on validating dataset of models with transfer learning weights from APTOS2019 Conv4, ImageNet Conv4, and CheXNet full train.

Performance indicators	APTOS2019 Conv4	ImageNet Conv4	CheXNet Full train
Sensitivity (recall)	0.97	0.95	0.92
Specificity	1.00	1.00	1.00
Positive predictive value (PPV, precision)	1.00	1.00	1.00
F1-score	0.99	0.98	0.96
Accuracy	0.98	0.97	0.95

*Conv* convolutional block, *APTOS* the Asia Pacific Tele-Ophthalmology Society.

**Table 4:** Performance indicators on testing dataset of models with transfer learning weights from APTOS2019 Conv4, ImageNet Conv4, and CheXNet full train.

Performance indicators	APTOS2019 Conv4	ImageNet Conv4	CheXNet Full train
Sensitivity (recall)	0.98	1.00	0.96
Specificity	1.00	0.98	1.00
Positive predictive value (PPV, precision)	1.00	0.98	1.00
F1-score	0.99	0.99	0.98
Accuracy	0.99	0.99	0.98

*Conv* convolutional block, *APTOS* the Asia Pacific Tele-Ophthalmology Society.

## Discussion

Our study demonstrated that the DenseNet121-based deep learning algorithm can successfully differentiate CMVR from normal retinal images. With different TL strategies, the mean accuracy reached between 0.91 and 0.99, while the mean loss fell between 0.04 and 0.43. The model with sequential transfer learning from the natural image dataset to another retinal image dataset before transferring the pre-trained weights to our dataset showed the best performance on both validating and testing steps. The sensitivity was as high as 0.99, and the specificity and positive predictive value were 1.00 on the testing dataset. The performance of our model exceeded those of the previous reports from Thailand and China^15,16^.

In CNN image analysis, TL, transferring feature weights trained on large image datasets to training on smaller datasets, is beneficial in developing a model on limited resources. The images of the target domain can be reduced to a significantly smaller number. The common practice is to use pre-trained weights from ImageNet, a large natural image dataset, and then train the model on the smaller target dataset as feature extraction or fine-tuning depending on the sizes and similarity of the target domain. However, the model performance may be unappreciated when the source and target domains have considerable dissimilarity.

Our study showed that sequential TL from natural to DR images was more favourable than conventional TL for a very small dataset like CMVR images. The weights from sequential training, from millions of images in ImageNet to thousands of DR images, APTOS2019, provided the best performance with statistical significance in TL to hundreds of CMVR images. This strategy improved the accuracy compared to the common practice, which directly uses pre-trained weights from ImageNet (0.9911 vs 0.9822, p = 0.02). On the other hand, sequential TL from natural to chest X-ray images was unrewarding, as seen in our model with pre-trained CheXNet weights.

In medical image analysis, the target domain differs remarkably from natural images such as persistent colours and anatomical correlation^31,32^. Therefore, the use of pre-trained weights directly from ImageNet to such a small dataset of CMVR images may not be appropriate. Sequential transfer learning by domain adaptation displayed benefits on TL from ImageNet to chest X-ray image classification^20^. Our results confirmed that 2-step sequential transfer learning from natural images (source domain) to other retinal images (intermediate domain) before training on our CMVR images (target domain) expressed the best strategy.

Moreover, some other interesting points are worth mentioning, including fine-tuning depth, performance scores, and class activation heatmaps. Our best model from APTOS2019 pre-trained weights demonstrated high performance and explainable heat maps while using fewer computation resources.

Feature extraction and fine-tuning are a spectrum of TL strategies. When the target dataset is small and different from the source dataset, the common recommendation is to freeze the shallow layers and fine-tune the model from the deeper layers of the convolution layer. When the deeper layers are frozen, the less computation resources are used. Our experiment showed that models with pre-trained weights from ImageNet and APTOS2019 performed best when we froze to the depth of convolutional block 4 of DenseNet121, which is just one block before the last, resulting in less computation consumed. In contrast, we needed to train a full model with feature weights from CheXNet. This finding also indicated that CMVR images had more similarity to diabetic retinal images and natural images, which were full-colour, than monochromatic images like chest X-rays. Although the retinal and chest X-ray images are both spatially preserved, colour space may play more critical roles in TL.

Our performances from the best three models on the validating datasets demonstrated specificities and positive predictive values of 1.00, indicating that all three models performed excellent in identifying normal retinal images and no misclassification of normal to CMVR images (false positive). Therefore, it ensures that we will not miss CMVR in the screening program when we use our model in practice. However, the model with pre-trained APTOS2019 weights had the best sensitivity among the three models, revealing that the fewest CMVR images were misclassified as normal (false negative). As a result, this model is the most suitable for CMVR screening programs. All models' performances improved on the testing datasets. The indicators from models with pre-trained APTOS2019 and ImageNet weights were comparable and still higher than the model with ChxNet weights.

The class activation heatmap is an important tool for describing the logic behind Deep Learning. Our model with pre-trained weights from the APTOS2019 dataset did not only demonstrate the best performance in diagnosing CMVR, but it was also excellent in visualizing the hot spots of CMVR lesions. The findings were similar whether executed on validating or testing image sets. Compared with two other models with different pre-trained weights, the model with APTOS2019 pre-trained weights highlighted lesions correctly for both CMVR lesions and normal retina, similar to the way clinicians interpret the lesions. The heatmaps covered more than 90% of the expected CMVR lesions while becoming more generalized in normal retinal images, as seen in Fig. 4a1–d1. These results explained the highest classification accuracy of the model.

In contrast, the model with ImageNet pre-trained weights covered more areas of CMVR lesions but missed the classification of the normal retina (Fig. 4a2–d2). This model may be more beneficial for future applications in automatic lesion segmentation. However, it could not identify normal fundus correctly. Unlike others, heatmaps from the model with CheXNet pre-trained weights could not explain the lesions appropriately for both CMVR and normal retina. Although it properly classified the groups, the heatmaps scattered and identified parts of CMVR incorrectly, as shown in Fig. 4a3–d3.

From our findings, although the classification performances were high among pre-trained weights from ImageNet, APTOS2019, and Chest X-ray14 datasets, the activated areas were different, as shown in activation heatmaps. TL from APTOS2019 pre-trained weights indicated more precise locations to detect CMVR lesions and normal areas than the other two models. This result supports the report from MRH Taher et al. that sequential training helps to improve the model performance.

There are advantages in the application of our model. Since our model is created based on retinal photos taken from the conventional retinal camera, it can be applied in local rural hospitals, which commonly have this type of camera. Our dataset was not fixated on only the central retinal area. Therefore, the application can be used on any part of the retinal fundus photographs. However, there were a few limitations in our study. First, our dataset consisted only of CMVR and normal retina. The performances may drop when the model is applied in real settings where the dataset may be mixed with other retinal diseases. Nevertheless, in the clinical setting, only the patients with clinical risks of CMVR are sent for CMVR screening. Therefore, the contaminations of other retinal diseases are less likely. Second, the performances were evaluated on the validation dataset. The performances in real clinical settings need to be explored in future experiments.

In conclusion, our study demonstrates that sequential transfer learning enhances model performance in CMVR diagnosis and underscores its potential to revolutionise screening practices in ophthalmology. By effectively utilising transfer weights from ImageNet to diabetic retinopathy images and then to the CMVR dataset, our approach improves accuracy and reduces computational demands. This advancement is particularly significant in resource-limited settings, offering a scalable and efficient solution for early detection of CMVR. Furthermore, our findings lay the groundwork for future research in automated disease diagnosis, potentially extending beyond ophthalmology to other areas of medicine where similar challenges exist. Future experiments on different CNN models and more classification groups will support the implementation of sequential transfer learning.

## Data Availability

All unidentifiable data are available at the corresponding author upon reasonable request.

## References

[1] Ford N, Shubber Z, Saranchuk P (2013). Burden of HIV-related cytomegalovirus retinitis in resource-limited settings: A systematic review. Clin. Infect. Dis..

[2] Munro M, Yadavalli T, Fonteh C, Arfeen S, Lobo-Chan AM (2019). Cytomegalovirus retinitis in HIV and non-HIV individuals. Microorganisms..

[3] Whitley RJ, Jacobson MA, Friedberg DN (1998). Guidelines for the treatment of cytomegalovirus diseases in patients with AIDS in the era of potent antiretroviral therapy: Recommendations of an international panel, International AIDS Society-USA. Arch. Intern. Med..

[4] CMV retinitis in China and SE Asia: The way forward [editorial]. *BMC Infect. Dis*. **11**, 327 (2011). 10.1186/1471-2334-11-327 PMC3252309 22115187

[5] Heiden D, Tun N, Maningding E (2014). Training clinicians treating HIV to diagnose cytomegalovirus retinitis. Bull. World Health Organ..

[6] Tun N, London N, Kyaw MK (2011). CMV retinitis screening and treatment in a resource-poor setting: Three-year experience from a primary care HIV/AIDS programme in Myanmar. J. Int. AIDS Soc..

[7] Ausayakhun S, Skalet AH, Jirawison C (2011). Accuracy and reliability of telemedicine for diagnosis of cytomegalovirus retinitis. Am. J. Ophthalmol..

[8] Shah JM, Leo SW, Pan JC (2013). Telemedicine screening for cytomegalovirus retinitis using digital fundus photography. Telemed. J. E Health.

[9] Yen M, Ausayakhun S, Chen J (2014). Telemedicine diagnosis of cytomegalovirus retinitis by nonophthalmologists. JAMA Ophthalmol..

[10] Jirawison C, Yen M, Leenasirimakul P (2015). Telemedicine screening for cytomegalovirus retinitis at the point of care for human immunodeficiency virus infection. JAMA Ophthalmol..

[11] Kingkosol, P., Pooprasert, P., Choopong, P., Hunchangsith, B., Laksanaphuk, V. & Tantibundhit, C. Automated cytomegalovirus retinitis screening in fundus images. In *42nd Annual International Conference of the IEEE Engineering in Medicine & Biology Society* (*EMBC*). 20201996 (2002). 10.1109/EMBC44109.2020.9175461 33018395

[12] Anwar SM, Majid M, Qayyum A, Awais M, Alnowami M, Khan MK (2018). Medical image analysis using convolutional neural networks: A review. J. Med. Syst..

[13] Ting DSW, Pasquale LR, Peng L (2019). Artificial intelligence and deep learning in ophthalmology. Br. J. Ophthalmol..

[14] Ting DSW, Peng L, Varadarajan AV (2019). Deep learning in ophthalmology: The technical and clinical considerations. Prog. Retin. Eye Res..

[15] Srisuriyajan, P., Cheewaruangroj, N., Polpinit, P. & Laovirojjanakul, W. Cytomegalovirus retinitis screening using machine learning technology. *Retina* (2022) (publish ahead of print). 10.1097/IAE.0000000000003506 35436264

[16] Du, K.-F., Dong, L., Zhang, K. *et al*. Development and validation of a deep learning-based system for detection of AIDS-related cytomegalovirus retinitis in ultra-wide-field fundus images. *SSRN Electron. J*. (2021).

[17] Pan SJ, Yang Q (2010). A survey on transfer learning. IEEE Trans. Knowl. Data Eng..

[18] Morid MA, Borjali A, Del Fiol G (2021). A scoping review of transfer learning research on medical image analysis using ImageNet. Comput. Biol. Med..

[19] Kim HE, Cosa-Linan A, Santhanam N, Jannesari M, Maros ME, Ganslandt T (2022). Transfer learning for medical image classification: A literature review. BMC Med. Imaging.

[20] Hosseinzadeh Taher MR, Haghighi F, Feng R, Gotway MB, Liang J (2021). A systematic benchmarking analysis of transfer learning for medical image analysis. Domain Adapt. Represent. Transf. Afford Healthc. AI Resour. Divers. Glob. Health.

[21] Wen Y, Chen L, Deng Y, Zhou C (2021). Rethinking pre-training on medical imaging. J. Vis. Commun. Image Represent..

[22] Huang, G., Liu, Z., Van Der Maaten, L. & Weinberger, K.Q. Densely connected convolutional networks. In *2017 IEEE Conference on Computer Vision and Pattern Recognition (CVPR). Proceedings of the IEEE Conference on Computer Vision and Pattern Recognition*. 4700–4708 (2017).

[23] Guillaumin, M. & Ferrari, V. Large-scale knowledge transfer for object localization in ImageNet. In *2012 IEEE Conference on Computer Vision and Pattern Recognition* (2012).

[24] *Applications K. DenseNet*. Accessed 16 Jan 2024 (2024).

[25] Society APT-O. *APTOS 2019 Blindness Detection Kaggle*. Accessed 9 May 2023 (2023).

[26] Rohith, R. *APTOS 2019: Keras Baseline Kaggle*. Accessed 16 Jan 2024 (2024).

[27] Rajpurkar, P., Irvin, J., Zhu, K. *et al*. *CheXNet: Radiologist-Level Pneumonia Detection on Chest X-Rays with Deep Learning*. cs.CV (2017).

[28] Wang, X., Peng, Y., Lu, L., Lu, Z., Bagheri, M. & Summers, R.M. Chestx-ray8: Hospital-scale chest x-ray database and benchmarks on weakly-supervised classification and localization of common thorax diseases. In *Proceedings of the IEEE Conference on Computer Vision and Pattern Recognition*. 2097–2106 (2017).

[29] Chou, B. *GitHub—Brucechou1983/CheXNet-Keras: This Project is a Tool to Build CheXNet-Like Models, Written in Keras*. Accessed16 Jan 2024 (2024).

[30] Zhou, B., Khosla, A., Lapedriza, A., Oliva, A. & Torralba, A. Learning deep features for discriminative localization. In *Proceedings of the IEEE Conference on Computer Vision and Pattern Recognition*. 2921–2929 (2016).

[31] Haghighi F, Taher MRH, Zhou Z, Gotway MB, Liang J (2021). Transferable visual words: Exploiting the semantics of anatomical patterns for self-supervised learning. IEEE Trans. Med. Imaging.

[32] Haghighi, F., Hosseinzadeh Taher, M.R., Zhou, Z., Gotway, M.B. & Liang, J. Learning semantics-enriched representation via self-discovery, self-classification, and self-restoration. In: *Medical Image Computing and Computer Assisted Intervention—MICCAI 2020: 23rd International Conference, Lima, Peru, October 4–8, 2020, Proceedings, Part I 23*. 137–147 (2020). 10.1007/978-3-030-59710-8_14 PMC9187496 35695848

